# Provenance in bioinformatics workflows

**DOI:** 10.1186/1471-2105-14-S11-S6

**Published:** 2013-11-04

**Authors:** Renato de Paula, Maristela Holanda, Luciana SA Gomes, Sergio Lifschitz, Maria Emilia MT Walter

**Affiliations:** 1 Department of Computer Science, University of Brasilia - UnB, Brasilia, Brazil; 2 Department of Informatics, Pontificial Catholic University - PUC/RJ, Rio de Janeiro, Brazil

## Abstract

In this work, we used the PROV-DM model to manage data provenance in workflows of genome projects. This provenance model allows the storage of details of one workflow execution, e.g., raw and produced data and computational tools, their versions and parameters. Using this model, biologists can access details of one particular execution of a workflow, compare results produced by different executions, and plan new experiments more efficiently. In addition to this, a provenance simulator was created, which facilitates the inclusion of provenance data of one genome project workflow execution. Finally, we discuss one case study, which aims to identify genes involved in specific metabolic pathways of *Bacillus cereus*, as well as to compare this isolate with other phylogenetic related bacteria from the Bacillus group. *B. cereus *is an extremophilic bacteria, collected in warm water in the Midwestern Region of Brazil, its DNA samples having been sequenced with an NGS machine.

## Introduction

The speed and efficiency with which scientific workflows may be performed have increased with the use of modern hardware and software technologies. One workflow can be executed many times with different programs, versions or parameters, or even modified input data, and scientists can compare results from these executions, which improves accuracy in data analysis. However, dealing with large volumes of information produced by many executions under a variety of conditions becomes increasingly difficult. In this context, new tools have to be developed to store data generated in each execution, together with the origin of this data and the details of a particular execution.

Therefore, on the one hand, data provenance is essential to scientific environments. Many works describe details of data provenance [1,2], classify their characteristics [3], propose models [4-7] and present practical applications [8,9]. Buneman et al. [10] define data provenance as *"the description of the origins of a piece of data and the process by which it arrived in a database"*. In other words, the origin of data used as raw material as well as the processes that transformed this data into the final product, must be identified and stored. It is important to note that, before choosing which data has to be stored, it is necessary to define how these data have to be structured so that they can be later recovered and understood. This explains the development of many distinct provenance models, e.g., W7 Model [4], Provenir Ontology [11], Provenance Vocabulary [5], Open Provenance Model (OPM) [6] and PROV-DM model [7].

On the other hand, genome projects aim to analyze DNA or RNA sequences produced by one NGS sequencer, e.g., 454/Roche [12]. According to the objectives of each project, many databases and programs are sequentially executed in a computational workflow, using DNA or RNA sequences as raw data and producing a large volume of information (Terabytes of data). Therefore, biologists analyze the generated data and, in order to obtain better results, propose improvements to the project, choosing different programs or requiring new executions of the same programs with different parameters. Thus, when one workflow execution is concluded, much information can be lost if there is no systematic procedure exists to store details of each execution, including adopted software and parameters, problems and related solutions, as well as information produced at each workflow step.

In this study, we propose to manage data provenance in bioinformatics workflows using the PROV-DM model [7]. This provenance model aims to store details of each workflow execution in a way that biologists can compare information generated among different executions and more efficiently plan new ones. Stored data includes raw and produced data at each workflow step, used parameters, the order in which the programs are executed and details of how data are linked. Our provenance model allows us to recover the process flow as well as to reconstruct the relations among input data and the processes generating new data, using different granularities.

## PROV-DM model

The PROV-DM model [7], proposed by the W3C (World Wide Web Consortium), is a conceptual data model that forms the basis for the W3C provenance (PROV) family of specifications. PROV-DM is based on the Open Provenance Model (OPM) [6] although more precisely defined. A PROV-DM showing the provenance description is graphically illustrated by a directed graph, rooted at the entity for which we want to explain the provenance.

**Figure 1:**
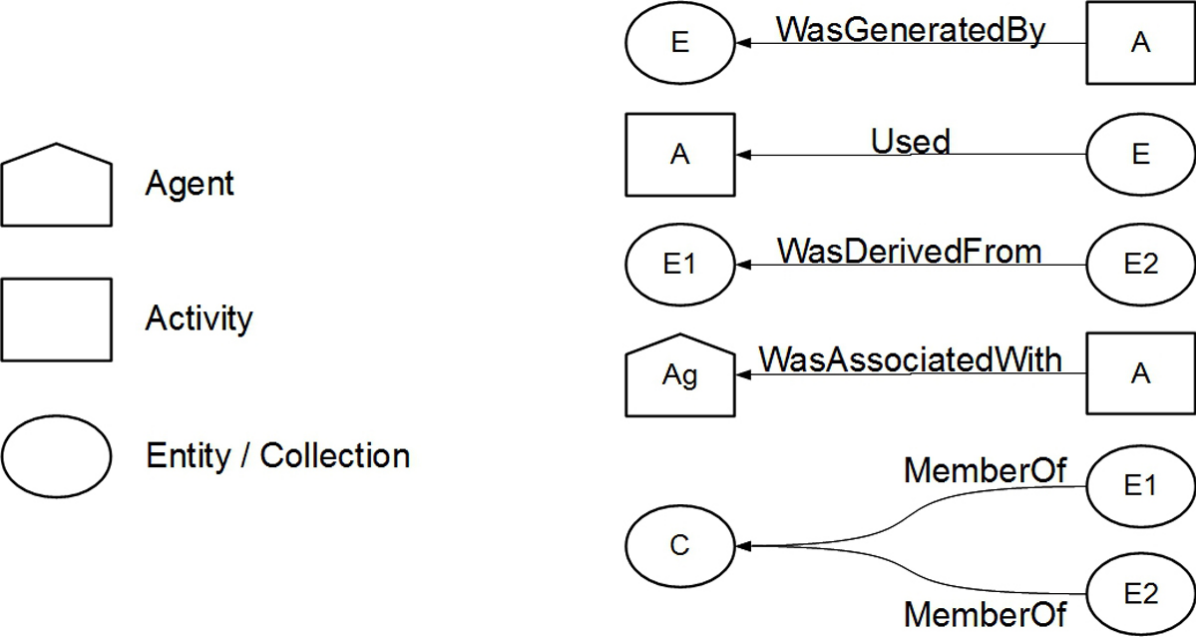
**PROV-DM elements**. Graphic representation of the elements *Agent*, *Activity *and *Entity *in the PROV-DM model.

PROV-DM core structure defines two initial elements, *Activity *and *Entity*, which can originate nodes in the provenance graph. *Activity *represents a process indicating the origin of one provenance object. *Entity *models any object representing some type of provenance. *Entity *element has two subtypes. The first one, *Agent*, can act over an Activity, or it presents some kind of responsibility over an Entity, e.g., owner or author's rights. The second one, *Collection*, represents a set of Entities, each one independent of its provenance content. Symbols representing elements of the provenance graph are illustrated in Figure 1.

Relations among the nodes are represented by the PROV-DM graph edges, which describesdifferent types of relations, the following ones being the most important to this study:

• *wasDerivedFrom *indicates that an original *Entity *was used, directly or indirectly, to generate another derived *Entity*;

• *wasQuotedFrom *is a particular type of wasDerivedFrom relation, and indicates that an Entity was generated from copying a part of an original *Entity*;

• *used *indicates that an *Entity *was used by an *Activity*;

• *wasGeneratedBy *indicates that an *Entity *was generated by an *Activity*;

• *memberOf *indicates which *Entities *belong to a *Collection*;

• *wasAssociatedWith *indicates that an *Activity *was associated to an *Agent*.

*Constraint *is another important PROV-DM property. Constraints are related to rules or restrictions associated with the construction of a provenance graph, having two main objectives: preventing the creation of invalid or inconsistent graphs; and making inferences about the elements and the relationships among them.

Besides this, PROV-DM *annotation *allows one to write additional information to any element of a provenance graph, which is extremely important since details about a specific process or element in a particular execution of a workflow can then be stored.

## Genome project

High-throughput sequencing machines, e.g., 454/Roche [12], produce hundreds of thousands of DNA or RNA fragments that have to be analyzed in genome or transcriptome projects. These NGS sequencers generate Gigabyte files, containing DNA or RNA sequences to be processed in computational systems named "workflows". Common phases in a workflow are described next.

In the *filtering *phase, the sequences are "cleaned" such that only the ones with a minimum of quality are included in the following phases, i.e., low quality sequences are discarded. This is possible because NGS sequencers provide quality for each base (represented as a letter in the alphabet Σ = {*A*, *C*, *G*, *T *(DNA) or *U *(RNA)}) of each fragment (a string in Σ). Data format generated by the sequencer is modified to string files, which will be processed in the following phases.

In the *assembly *phase, groups of fragments sharing similar regions are clustered into *contigs*. This is done for large fragments, such as those generated by 454/Roche. Longer regions containing contigs form *scaffolds*, constructed using a phylogenetic related organism as reference genome.

In the *analysis *phase, according to the objectives of the genome or transcriptome project, different analyses may be executed, e.g., annotation (a biological function is assigned to each contig, using comparison algorithms and large databases), identification of enzymes in order to find putative metabolic pathways involved in certain reactions, or phylogenetic reconstruction showing evolutionary relationships among the studied and related organisms. The analysis phase is the most space expensive one, since it may generate Terabytes of information, when compared to the previous phases, which generate Gigabytes of information.

In a genome or a transcriptome project, information may be stored in a file system or a DBMS. Input (raw data) for the workflow is the set of sequences generated by one NGS sequencer. As previously mentioned, outputs depend on each project objectives, but each workflow phase deals with specific databases and programs generating very large amounts of data. This information, together with versions and parameters of the programs, must be stored. Moreover, genome and transcriptome projects usually involve different executions of the same workflow using different parameters or including other databases. In this context, a provenance model is very important since it guarantees access to details of each execution of the workflow, and this allows the biologists to study how the modifications can affect the resulting data, so improving the analyses.

## Data provenance in genome projects

Managing data provenance in genome projects is essential to control how the enormous volumes of data were generated since, as mentioned earlier, they involve distinct analyses performed by many programs and data bases, with different versions and parameters. We propose here to use the PROV-DM model to manage data provenance in bioinformatics workflows. The PROV-DM model is simple, well documented, and can be easily adapted to include provenance in genome workflows [13].

We propose a model with two levels of granularity, in order to keep structured bioinformatics provenance information. The first level corresponds to the PROV-DM model based provenance graph, and includes details about each graph node. The graph is composed by *Collections*, performed *Activities *and *Agents *operating in a particular execution, besides presenting relationships between each pair of elements. *Collections *are used to represent a group of *Entities*, which are composed of millions of DNA or RNA sequences.

Relations in our PROV-DM based model are:

• *memberOf *linking *Entities *to *Collections*;

• *Used *and *WasGeneratedBy *maintain their behavior when *Collection *is used;

• *WasDerivedFrom *characterizes a transformation of an *Entity *or a *Collection *into another.

To represent data provenance in a bioinformatics workflow, we propose to include two new elements, *Project *and *Workflow Execution*, besides the PROV-DM components. *Project *was created to join distinct re-executions of a genome project workflow. *Workflow Execution *stores information about one workflow execution. A set of minimum information related to each entity is shown in Table 1.

**Table 1:** Information of the provenance graph for a genome project (already shown in de Paula [16])

PROJECT		WORKFLOW EXECUTION	
Name Description Funding Institutions Partner Institutions Coordinator Start Date End Date	Name of the *Project* Description of the *Project* List of the *Project *funding institutions List of the *Project *partner institutions Name of the *Project *coordinator *Project *Start date *Project *End date	Name Description Location Date Version Date Version Notes	Name of the *Workflow Execution* Description of the *Workflow Execution* Execution Location Execution Date Version Date Version number Any information about the execution of the *Workflow Execution*

**AGENT**		**ACTIVITY**	

Name Institution Position Function Groups Notes	Name of the person Affiliated Institution Position or Function Function in the *Workflow Execution* Filtering provenance graph groups Any information about *Agent*	Name Program Version Command Line Function Start Time End Time Environment Groups Notes	Name of the *Activity* Program Name Program Version Command Line used and parameters Description of what is being done Date and time the *Activity *began Date and time the *Activity *ended Description of computational environment Filtering provenance graph groups Any information about the execution of the *Activity*

**COLLECTION**		**ENTITY**	

Name Size Description Location Group Notes	Name of the *Collection* Number of *Entities *contained in *Collection* Description of the *Collection *content Location of the file or database with the Content of the *Collection* Filtering provenance graph groups Any information about the content of the *Collection*	Name Description Location Group Notes	Name of the *Collection* Description of the *Entity *content Location of the file or database with the content of the *Entity* Filtering provenance graph groups Any information about the content of the *Entity*

The second level allows access to the original input files used in one workflow execution. e.g., transcripts (expressed genes), as well as the output files produced in each phase of the workflow. Since one execution of a workflow generates large amounts of information, which may, or may not, be relevant for future analysis, biologists can decide what information will be stored or discarded.

Biologists can choose which levels will be maintained. The first level needs only a few Bytes of memory since it requests little information, in contrast to the second level that may require Terabytes of space, depending on the genome project objectives. Although separated, information of the two levels remain linked, which allows transparent access to these data.

## PROV-DM for provenance in bioinformatics workflows

PROV-DM model defines elements and relationships, as well as restrictions and inferences that can be used in a variety of ways according to the context in which data provenance is applied. In this section, these topics are discussed with regard to bioinformatics workflows.

### Managing elements

The PROV-DM elements used in this project are *entity*, *activity*, *agent *and *collection*, which represent possible nodes of the provenance graph, and *account *to represent the graph itself. In the following, we discuss how we used them in our proposal of a provenance model for bioinformatics workflows.

We first address the *entity *element, which has three types: *agent*, *account *and *collection*. The *agent *element will be used to represent any person, institution or service that makes some kind of action in an activity. The *account *element will capture the execution of a particular experiment, and therefore it represents a provenance graph. The *entity *element will be used to represent basic data, e.g., a DNA sequence or an alignment. When a workflow is executed in a genome project, the *entity *element will be used when the user needs to know the content of a *collection*. It is always possible to graphically exhibit all the entities, but since some information, such as the raw data, wastes large amounts of storage space, sometimes it is not possible to show all of them, but only the collections themselves.

The other basic PROV-DM element is *activity*. In bioinformatics projects, an *activity *represents any process that can be executed in an experiment (workflow). The *activity *element will indicate the properties of one executed program, including command lines and program name and version, among others. Notably, among the elements composing the graph (*entity*, *agent*, *collection *and *activity*), the later is the only one having temporal characteristics (start and end times).

Finally, with the objective of improving the creation of personalized provenance graphs based on distinct executions of a workflow, an attribute called *Group *was defined. This attribute can be found in the nodes of the graph or in the *collection *elements. A group is defined by the user when each element is created and, due to its multiple attribute status, each element can be part of different groups. This allows the user to visualize only a part of the provenance graph by choosing some of these groups.

### Managing relations

PROV-DM relations are represented by edges linking different nodes of the provenance graph. These edges are directed and show how each object was generated, since the path in the graph begins in the generated object and comes to the origin of this data, passing through the events that generated that object. The PROV-DM model describes different types of relations, and since we are working on bioinformatics projects, we have adopted some definitions.

The first relation to be considered is *Used*, which links a collection or an entity to the activity that used this collection or entity. A directed edge goes from the activity to a collection or entity, which can be used in distinct activities. Similarly, each activity can use different collections or entities. This is good for bioinformatics projects, since a particular file can be used by different processes, e.g., a genome reference can be used in different mapping processes.

The relation *WasGeneratedBy *indicates which activity generated a particular collection or entity. The edge goes from the collection or entity to the activity that generated it. One activity can generate a variety of collections or activities, however each collection or entity can only be generated by a single activity. This way, different *WasGeneratedBy *relationships can be created for only one activity, however, only one activity can occur for each collection or entity. This definition models the processes executed in bioinformatics projects, where one particular data (or file) can only be generated by a single program.

The relation *WasAssociatedWith *stores the agent that has executed an action of an activity. The edge goes from the agent to the activity. Multiple connections among different agents and activities are allowed.

The relation *WasDerivedFrom *indicates how the link was derived, during the execution of one experiment, between the used and the generated data. In a bioinformatics workflow, different processes can be executed, so the attribute *Type of Derived Link *was associated to each edge with one of the following characteristics: *filtering*, the collection was derived by a filtering process; *ordering*, the derived collection was rearranged according to some ordering criterion; *mixing*, the created collection has a data format different from the original one; *other*, any other process distinct from the previous ones.

Considering that the relations *Used*, *WasGeneratedBy*, *WasAssociatedWith *and *WasDerivedFrom *can present multiple links, and when the identification of these links store important provenance information in the graph, the attribute *Role *provides the identification of each one of these links. This way, each of the four relationships has an associated attribute *Role*, which value will be defined by the user to precisely indicate the reason why the corresponding edge has been created.

### Restrictions

As previously stated, PROV-DM model defines a list of generic restrictions in order to allow the validation of the provenance graph. These restrictions are divided into three types: structural, temporal and functional, described as follows.

#### Structural restrictions

Structural restrictions allow the construction of a more concise provenance graph, avoiding ambiguous information and guaranteeing that the execution of each experiment (one execution of the bioinformatics workflow) can be reproduced. The most important restriction for keeping the provenance graph structured is that each element of the graph has to have a single identifier, including the graph itself. This also supports the creation of relationships using the identification of each element.

Another structural restriction is the creation of valid relationships in the provenance graph. A single relationship can be created from *existing *and *valid *nodes. *Existing *nodes are those that have already been created and inserted in the experiment, and *valid *nodes are those expected for each relationship.

These relationships allow the construction of the provenance graph structure. Thus, we defined a list of restrictions that must be followed when creating a relationship:

• no two identical relationships exist, i.e., it is not possible to have two relationships of the same type with the same origin and the same destination;

• only one relationship *WasGeneratedBy *linked to each entity or collection of the provenance graph is allowed;

• in the relationship *WasDerivedFrom*, the elements "origin" and "destination" must be different.

#### Temporal restrictions

The objective of temporal restrictions is to avoid creating graphs representing sequences of processes that are impossible to occur in a bioinformatics workflow. To simplify the creation of the bioinformatics workflow provenance graph, we defined three elements with temporal characteristics: project, account (representing the graph itself) and activity.

An activity models the execution of some process having a start time and an end time. This characteristic is passed to the other elements as follows: *WasGeneratedBy*, the entities and collections will have the hour in which the generated activity was completely concluded as the start time; *Used*, the entities and collections will have as their start times the hours that the used activity began as their start times; *WasAssociatedWith*, the agent activity times are the activity start time and end time.

These characteristics allowed us to adopt the following restrictions:

• *Activity*: the start time of an activity must be less than its end time;

• *WasGeneratedBy*: a collection or an entity can only be generated by an activity whose end time is less than all the hours when this collection or entity has begun to be used;

• *Used*: analogously, an activity can only use a collection or entity if the start time of this activity is greater than the start time of this collection or entity;

• *WasDerivedFrom*: a collection or entity can only be derived from another original collection or entity if the start time of the original is less than the hour of having generated the derived one.

**Figure 2:**
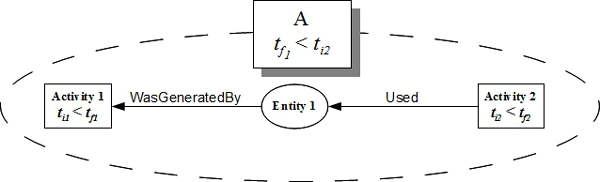
**A graph with a minimum set of elements allowing the verification of temporal restrictions**. Besides the *activity *element, two other elements have temporal characteristics, *account *and *project*. Both elements work only with the start time and end time. This way, four more restrictions can be defined: *Account*: the start time of an account must be less than or equal to the end time; *Account-Activity*: an account can contain only activities executed between start time and its own execution end times; *Project*: the start time of a project must be less than or equal to the end time; *Project-Account*: a project can only contains accounts representing experiments executed between the project start and end times.

Temporal restrictions of *Used*, *WasGeneratedBy *and *WasDerivedFrom *can only be evaluated when the elements that can be validated are identified, which means that the restrictions can only be observed from the moment that there exists at least one collection or one entity being used by an activity and being generated by another activity exists. This is a minimum set of temporal restrictions that need to be verified. Figure 2 shows an example of a graph having a minimum set of elements that allows the verification of these three temporal restrictions, since the temporal characteristics (start and end times of each activity) need to be validated.

#### Functional restrictions

The last type of restrictions refers to the functionality of an experiment. Collections point to sets of data that are usually files containing information used and generated during the execution of this experiment. This restriction refers to the correct identification of these collections. Thus, collections can only indicate sets of correct data, or rather, indicate a file that has to have its existence verified.

### Inferences

Inferences allow the verification of some *Roles*. In our provenance model, some inferences were determined from observing some restrictions described in the previous section.

The first inference is related to the structural validity of an account, which can be verified from the account structural restrictions. Once all of these structural restrictions are verified, one can infer that the account is structurally valid. Otherwise, this account can be inferred as structurally invalid. Analogously, the structural validity of a project can also be inferred. Therefore, if all the accounts belonging to a project are structurally valid, then this project is also inferred as structurally valid. If it is not the case, even if only one account is structurally invalid, the project is considered structurally invalid.

The temporal inference analyzes temporal characteristics of the account. Thus, we can verify those related to the account start and end times. If all these restrictions are correct, the other two situations related to the activity temporal restrictions must be followed. The temporal restrictions referring to *Used*, *WasGeneratedBy *and *WasDerivedFrom *relationships will only be possible if there is a minimum set of nodes and edges. For accounts not having a minimum set, this restriction will be automatically satisfied. Therefore, for an account to be inferred as temporally valid, the temporal restrictions of their start and end times have to be satisfied, as well as the temporal restrictions of its activities and edges. Otherwise, it is considered temporally invalid. A project, in its turn, can be inferred as temporally valid if the restrictions related to its start and end times are satisfied, and all the accounts that belong to this project are temporally valid. If this is not the case, the project is inferred as temporally invalid.

The functional inferences are related to the functional restrictions, thus one can infer that an experiment is functionally valid if all its collections point to sets of valid data. Otherwise, it will be inferred as functionally invalid. An experiment considered functionally invalid is not necessarily treated as an experiment containing errors, but it means that at least one of its collections points to some non-available data set. The user can choose to maintain or not some files used in an experiment and, if a file is deleted from this experiment, it will be functionally invalid. Even though it may not be considered an error, the evaluation of the functional validity is important, since it means that this experiment cannot be re-executed due to a possible lack of necessary elements. Analogously, a project will be considered functionally valid if all of its experiments are functionally valid. If one experiment is considered functionally invalid, then the entire project is considered functionally invalid.

## Related work

Some projects in the literature propose to store provenance data in Database Management Systems (DBMS). The DBMS was used in the proposals of Jones et al. [14], Mungall and Emmert [15] and Paula et al. [16], which created biological databases with specific modules to include provenance data. However, the DBMS table structure is inflexible to store the different types of genome projects data.

Other projects use the W7 model. Marins et al. [17] present an application to find information in personal computers, and catalog them to facilitate their research. Liu and Ram [18] use provenance data to evaluate the quality of Wikipedia pages based on the ways users collaborate. Orlandi et al. [9] present a model using pages and categories for provenance capture in Wikipedia pages to show user contribution information. The Provenance Vocabulary model [19] presents an application that uses provenance data on the web for evaluating the quality of available information. Omitola et al. [20] extends this model to integrate web data. Kessler et al. [21] treat provenance data in OpenStreetMap using the Provenance Vocabulary model.

Provenir Ontology [22] extends a model designed to deal with biological data in a project having the objectives of developing a vaccine, a diagnostic exam and a chemotherapy treatment for the *Trypanossoma cruzi *human pathogen. Missier and Belhajjame [23] use the Provenir Ontology model together with a module for treating biological data, addressing the data collection generated by Taverna (one Scientific Workflow Management System - SWfMS) [24]. As an example, they discuss how to search known relationships between a specific region in the mouse genome, known as QTLO (Quantitative Trait *Loci*), and the metabolic pathways involving genes located in this region. Patni et al. [25] present a framework to store and query provenance data in meteorological data.

OPM is the most widely used model in applications of provenance now, as can be seen by the variety of projects using it. Here we discuss some of them. Cao et al. [26] present an OPM based system to capture and generate provenance data in scientific experiments. Marinho et al. [27] and Coutinho et al. [28] aim at managing provenance information in heterogeneous and distributed environments using OPM. Chapman et al. [29] define an OPM based model to evaluate data reliability using provenance information. Braun et al. [30] evaluate the interoperability of provenance data among systems PASS (Harvard Provenance Aware Storage System) and MITRE's PLUS. Gomes [31,32] defines an OPM based system for capturing provenance data from biological workflows using SWfMS, storing these data in a database modelled according to the OPM model.

As far as we know, there are no applications based on PROV-DM for bioinformatics applications, since it is a very recent model. However, it is noteworthy that Missier and Belhajjame [33] present an application of deduction rules to verify the validity of provenance graphs.

Our model was designed to store and manage provenance data independent of any SWfMS, e.g., *ad-hoc *projects. In fact, little additional information is included in the original files in order to retrieve information using specific modules directly linking biological data types in these files. Our model does not address automatic capture of provenance data.

## Case study

In this section, we first describe our Provenance simulator, and subsequently discuss one case study.

### The Provenance simulator

A Provenance simulator was implemented in Java (details shown in de Paula [16]). We used two external libraries. The first one, OPM4J [6], was used to create an OPM based provenance graph. This library was used due to its stability, and to the fact that PROV-DM and OPM are very similar, and PROV-DM does not have completely developed libraries yet. The second one, GraphViz [34], was used to visualize the created provenance graph.

The simulator has a graphic interface allowing the user to inform provenance data, besides storing data in XML files. The user can create or visualize the provenance graph at any time, when a workflow is executed. The elements of the XML schema correspond to the information described in Table 1, created for each *Project*. Besides, a list was generated with the name and location of the XML files representing each *Workflow Execution *performed in a particular *Project*.

**Figure 3:**
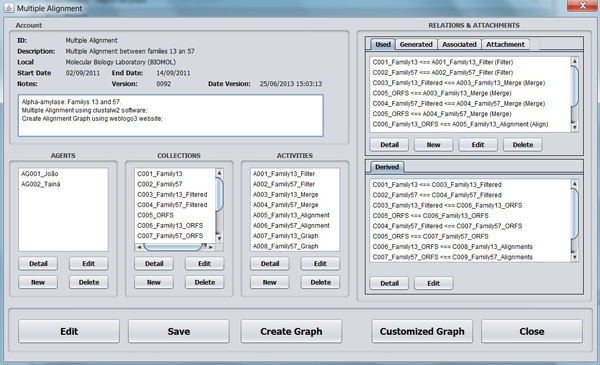
**Main frame of the Provenance simulator**. In the main frame of the simulator, called Provenance, a user can enter information to store data provenance for each execution of a particular bioinformatics workflow. In the upper left frame, general information of the execution (description and execution date) can be seen. Besides, elements *Agent*, *Entity *and *Activity *are listed, together with the three types of relations and the annotations called *Attachments*. With the objective of making it user friendly, *Collections *were implemented as *Entities *with "Size" property greater than 1. Finally, a relation *WasDerivedFrom *is found on the lower right frame, since it cannot be modified by users. Its creation or removal is originated from the creation/deletion of the relations *Used *and *WasGeneratedBy*.

**Figure 4:**
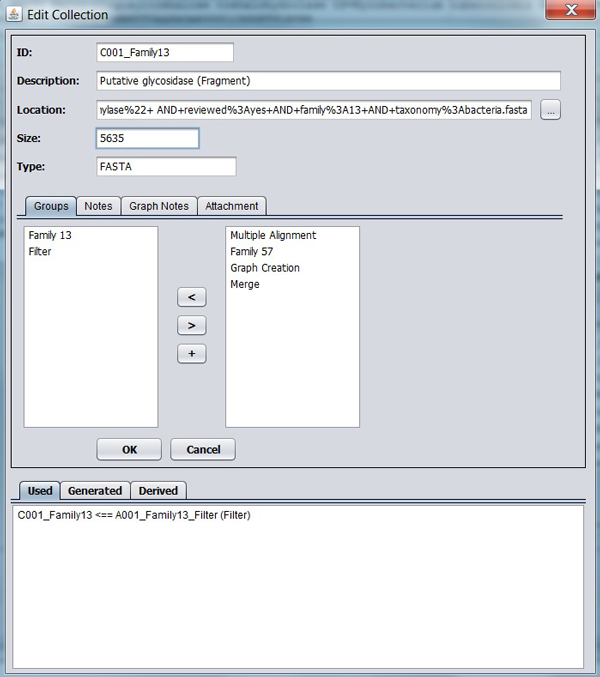
**Sample of Entity edition frame**. Data from C001_Family13, its groups and relations are shown in the frame data.

**Figure 5:**
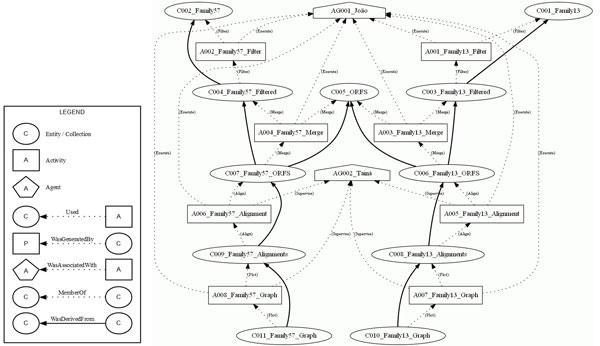
**The provenance graph generated from the *Bacillus cereus *genome project**. One of the objectives of this genome project is to compare the sequences of an isolate of the extremophilic bacteria *Bacillus cereus *with others belonging to related species in the Bacillus group. These comparisons were done using multiple alignments carried out for samples of the isolate alpha-amylase families 13 and 57, and drawing up graphics for these alignments. Data provenance of these comparisons are shown in the provenance graph executed with the workflow, composed of 2 agents, 11 collections and 8 activities linked by 40 edges.

The Provenance simulator is shown in Figure 3, where the case study *Multiple Alignment *is displayed using information of an XML file previously stored. To use this simulator, the user needs to create a project, enter provenance data (Table 1), and inform the graph nodes (*Agent*, *Activity *and *Entity*) and relations linking these nodes (Figure 4). Data is stored in XML files, noting that, whenever this file is saved, the simulator modifies the file version and date, and a *gif *format file containing the provenance graph is generated. The simulator allows to visualize the provenance graph, as shown in Figure 5.

### The project: Identifying metabolic pathways of alpha-amylases in a bacterial isolate

**Figure 6:**
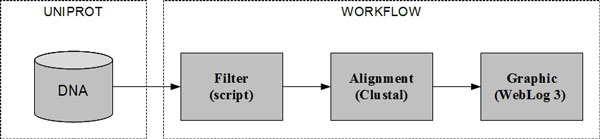
**Workflow for finding alpha-amylase genes of a Brazilian *Bacillus cereus *isolate**. The DNA sequences obtained from UNIPROT were first filtered and then aligned to a file containing the sequences of the alpha-amylase genes. Finally, the file with the multiple alignments was used to generate graphics.

This case study deals with the DNA sequencing of the extremophilic bacteria *Bacillus cereus*, of which its isolate was collected in warm water in a city of the MidWest Region of Brazil. The objective of this project is to find the genes codifying for the alpha-amylase (obtained from UNIPROT [35]) in the *B. cereus *genome, as well as to compare their sequences with others belonging to related species in the Bacillus group. In this case study, we modeled data provenance of the comparisons among the sequences found in this isolate with genes of the related organisms. These comparisons were done using multiple alignments carried out for samples of the isolate alpha-amylase families 13 and 57, and drawing up graphics for these alignments. This project is under development in the Molecular Biology Laboratory of the Department of Cellular Biology at the University of Brasilia (UnB). Figure 6 shows the workflow of this experiment, and Figure 5 shows the provenance graph generated from one execution of the workflow.

This experiment was executed by a student of the Department of Computer Science at UnB, modeled by the agent AG001_Joao, and was validated by a researcher from the Department of Cellular Biology, represented by the agent C002_Taina. Agent AG001_Joao is associated to all of the activities executed in this experiment, which is represented by the dotted edges between this agent and each activity on the graph. The edges represent the PROV-DM *WasAssociatedWith *relation. Along with this, the role of this agent was to execute each activity, which is shown by the *Execution *role present at each of its relationships. Agent C002_Taina validated four activities of the experiment, A005_Family13_Alignment, A006_Family57_Alignment, A007_Family13_Graphic and A008_Family57_Graphic, which is represented by the relationships among this agent and its activities, as well as by the role associated to each of these relationships.

Collections C0001_Family13 and C002_Family57 represent FASTA format files with the DNA sequences of families 13 and 57, respectively, of the alpha-amylases obtained from UNIPROT. Collection C0005_ORFS also represents a FASTA file of the putative proteins found on the contigs, which will comprise a part of the aligned ORFs in each family.

Initially, a filtering process was carried out, represented by activity A001_Family13_Filter, which used the original collection C001_Family13 to generate the filtered collection C003_Family13_Filtered. Next, the activity A003_Family13_Mix executed a process in order to join collections C003_Family13_Filtered and C005_ORFS, generating the collection C006_Family13_ORFS. After that, the collection C006_Family13_ORFS was used by the activity A005_Family13_Alignment with the purpose of arranging the multiple alignment of the sequences in this collection. These alignments were recorded in a file represented by the collection C008_Family13_Alignments. Finally, the collection C010_Family13_Graph was generated by the activity A007_Family13_Graph, which used the alignments contained in the collection C008_Family13_Alignments. The same processing steps occurred with the collections of Family 57, which generated the collection C011_Family57_Graph.

**Figure 7:**
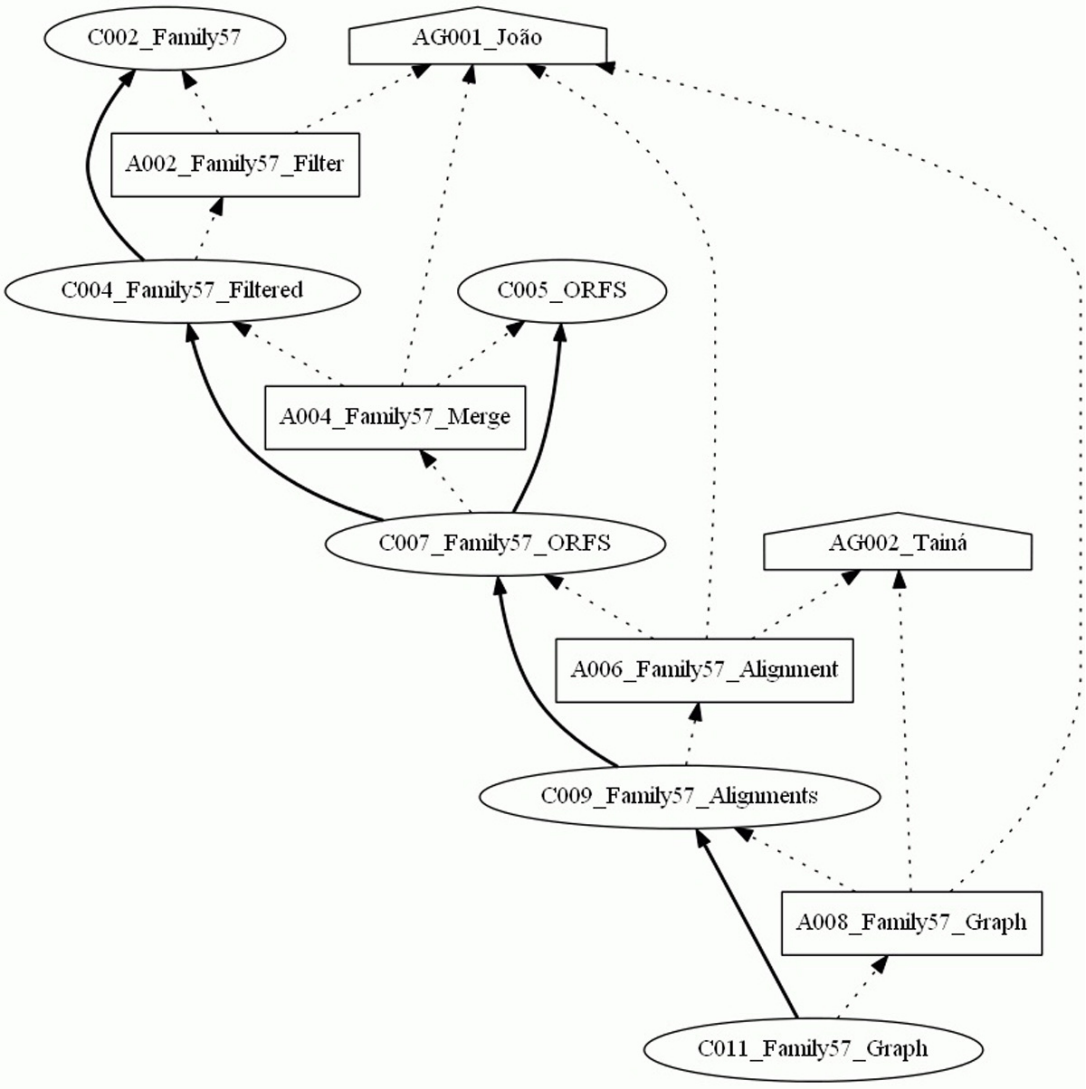
**The provenance graph of the Family 57 group**. This figure shows the provenance graph obtained when selecting the *group *Family 57, and not selecting the options to show the graph roles and annotations.

**Figure 8:**
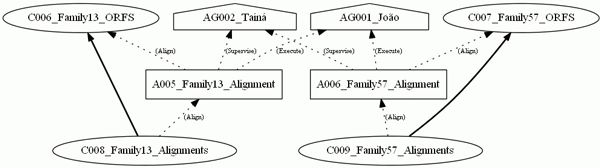
**The provenance graph of the ***Multiple Alignment ***group and ***roles*. This graph only shows the nodes belonging to the *Multiple Alignment *group and their respective *roles*.

**Figure 9:**
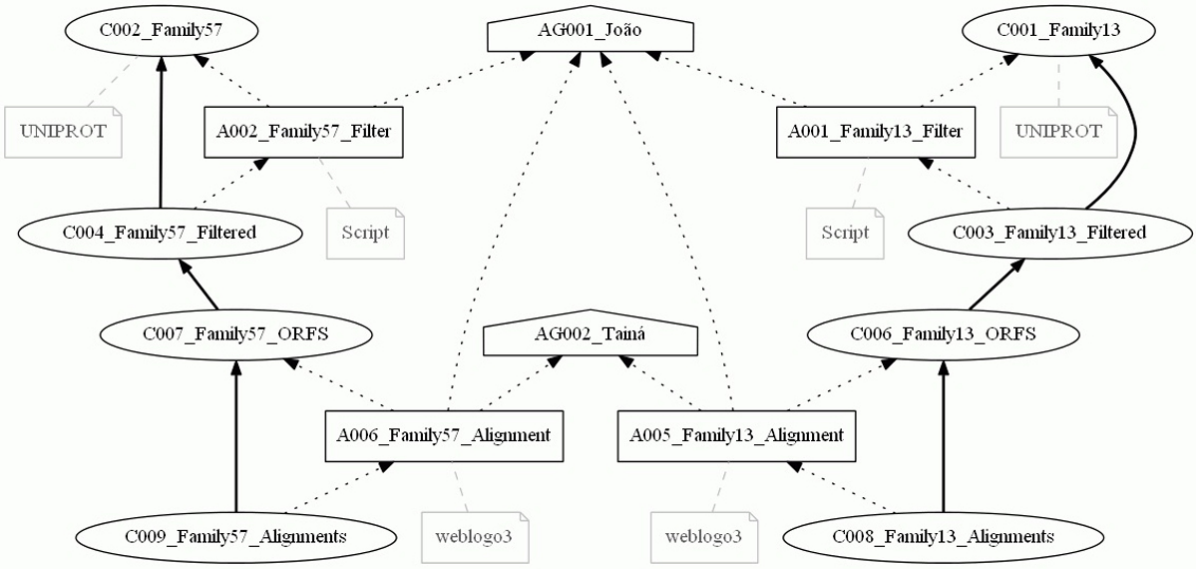
**The provenance graph with groups ***Filter ***or ***Multiple Alignment ***and annotations**. This graph was generated with nodes belonging to at least one of the groups, *Filter *or *Multiple Alignment*, and an option to show the annotations appearing in gray rectangles. Note that annotations can be included by the user at each node in the graph, but are not mandatory, e.g., agent AG001_Joao has no annotation.

With the defined element *group*, it was possible to select parts of the experiments. In Figure 7, the *group *Family 57 was the only one selected, noting that the options to show the graph roles and annotations were not selected. In Figure 8, the provenance graph included the *Multiple Alignment *group and the option to present *roles*. Therefore, the graph shows only the nodes belonging to the *Multiple Alignment *group and their respective roles. Finally, a provenance graph was generated with nodes belonging to at least one of the groups, *Filter *or *Multiple Alignment*, together with an option to show the annotations. Figure 9 shows the graph with nodes belonging to at least one of the chosen groups, with the annotations indicating the origin of some of the collections and programs used for the activity executions.

## Conclusion

In this work, we propose to use the PROV-DM model to manage data provenance in genome projects. The PROV-DM model allows to store the properties of each execution of a bioinformatics workflow. To graphically represent the large volumes of data generated at the genome projects, we used *Collections *of *Entities *to represent groups of large sets of data, and the same files storing the project data to construct the *Collections*. The proposed provenance model was divided in two levels, one corresponding to the provenance graph itself and the other providing access to the data of a particular execution. Biologists can investigate the entire or just a portion of the provenance graph.

We also developed one case study, including provenance in a bioinformatics workflow with real data. This experiment shows that little additional data has to be generated in order to maintain provenance. To create the provenance graph, we only need to connect data provenance to the files already used in a particular *workflow execution*. This case study was developed in order to show the usability of the provenance model in a real genome project, which had the objective of finding alpha-amylases in an isolate of an extremophilic bacillus collected in warm water in the MidWest Region of Brazil. We also created the Provenance simulator, for storing details of the *workflow execution *in XML files.

We are developing a module in the Provenance simulator to automatically capture provenance data from a *workflow execution*, with few information given by the user. Besides, it is useful to integrate our Provenance simulator in a bioinformatics workflow implemented using a SWfMS.

## Competing interests

The authors declare that they have no competing interests.

## Authors' contributions

R. P. implemented the Provenance simulator and developed the experiments. R. P., M. H. and S. L. discussed the experiments. All the authors proposed the provenance model and contributed to the text.

## Declaration

The publication costs for this article were funded by the University of Brasilia.

This article has been published as part of *BMC Bioinformatics *Volume 14 Supplement 11, 2013: Selected articles from The Second Workshop on Data Mining of Next-Generation Sequencing in conjunction with the 2012 IEEE International Conference on Bioinformatics and Biomedicine. The full contents of the supplement are available online at http://www.biomedcentral.com/bmcbioinformatics/supplements/14/S11.
